# The articles.ELM resource: simplifying access to protein linear motif literature by annotation, text-mining and classification

**DOI:** 10.1093/database/baaa040

**Published:** 2020-06-08

**Authors:** N Palopoli, J A Iserte, L B Chemes, C Marino-Buslje, G Parisi, T J Gibson, N E Davey

**Affiliations:** 1 Departamento de Ciencia y Tecnología, Universidad Nacional de Quilmes, CONICET, Roque Saenz Peña 352, Bernal, Buenos Aires B1876BXD, Argentina; 2 Fundación Instituto Leloir, Instituto de Investigaciones Bioquímicas de Buenos Aires, CONICET, Av. Patricias Argentinas 435, Ciudad de Buenos Aires C1405BWE, Argentina; 3 Instituto de Investigaciones Biotecnológicas, Universidad Nacional de General San Martín, IIB-INTECH-CONICET, Av. 25 de Mayo y Francia, San Martín, Buenos Aires B1650, Argentina; 4 Structural and Computational Biology Unit, European Molecular Biology Laboratory, Meyerhofstraße 1, Heidelberg 69117, Germany; 5 Division of Cancer Biology, The Institute of Cancer Research, 237 Fulham Road, London SW3 6JB, UK

## Abstract

Modern biology produces data at a staggering rate. Yet, much of these biological data is still isolated in the text, figures, tables and supplementary materials of articles. As a result, biological information created at great expense is significantly underutilised. The protein motif biology field does not have sufficient resources to curate the corpus of motif-related literature and, to date, only a fraction of the available articles have been curated. In this study, we develop a set of tools and a web resource, ‘articles.ELM’, to rapidly identify the motif literature articles pertinent to a researcher’s interest. At the core of the resource is a manually curated set of about 8000 motif-related articles. These articles are automatically annotated with a range of relevant biological data allowing in-depth search functionality. Machine-learning article classification is used to group articles based on their similarity to manually curated motif classes in the Eukaryotic Linear Motif resource. Articles can also be manually classified within the resource. The ‘articles.ELM’ resource permits the rapid and accurate discovery of relevant motif articles thereby improving the visibility of motif literature and simplifying the recovery of valuable biological insights sequestered within scientific articles. Consequently, this web resource removes a critical bottleneck in scientific productivity for the motif biology field. Database URL: http://slim.icr.ac.uk/articles/

## Introduction

The first definition of a short linear motif (also known as a SLiM, MoRF or miniMotif) was coined in 1990 in an announcement for a *Trends in Biochemical Sciences* article series titled ‘Protein sequence motifs involved in recognition and targeting: a new series’ (5). At the time, only a handful of SLiM-related articles had been published and less than 10 motif classes had been characterized, including the nuclear localization signal (NLS), the integrin-binding RGD motif and the KDEL endoplasmic reticulum retrieval motif (6,9,10). Despite the limited dataset, the definition included many of the key attributes of SLiMs such as their short length, physicochemical degeneracy and absence of tertiary structure (3). Almost 30 years later, our understanding of SLiMs has exploded, revealing a central role in all aspects of cell regulation (7,14,17). SLiMs have been shown to play a particularly important part in the regulation of protein half-life, localization and modification state (7). Consequently, the motif literature provides an invaluable record of the complex decision-making taking place in the higher eukaryotic cell (15,16).

Unfortunately, much of the characterized data on SLiMs is not readily accessible. As motif biology straddles several biological fields, and much of the motif terminology is not standardized, many of these articles are invisible to typical search strategies. Curation and organization of the available literature can help overcome this issue. However, curation of protein motif information from the literature is a time-consuming process. Over the past 15 years the Eukaryotic Linear Motif (ELM) resource (7), the gold standard database of SLiMs, has curated about 3000 motifs in approximately 2000 papers and classified these motifs to about 285 motif classes. Still, at least 6000 additional articles related to SLiMs await curation by the ELM resource. Many of these articles describe novel motif classes that have not been curated in the ELM resource. Based on the uncurated articles, we estimate that greater than 600 motif classes have been described experimentally and the curation of the available literature would double the number of classes in the ELM database. In this manuscript, we introduce the articles.ELM repository, a companion for the ELM resource. The resource contains a manually collected compendium of about 8000 motif related articles. The articles.ELM repository represents a resource for deposition, exploration, annotation and classification of the protein motif literature corpus. The repository provides a simple mechanism to access the research output of the motif biology community that is hidden within the text of scientific articles, thereby allowing wider dissemination of motif information.

The resource has four major roles, functioning as (i) a repository for published articles related to protein motifs, (ii) a searchable interface for these articles, (iii) a tool to classify and rank these articles based on their similarity to the manually curated motif classes in the ELM resource and (iv) an interface to manually classify these articles. At the core of the framework are two article annotation tools. Firstly, all articles are partially curated with protein annotation using the available article-linked protein data across a range of biological repositories of manually curated protein information. This simplifies motif literature searches by annotating motif articles with protein metadata. Secondly, machine learning tools automatically classify articles to motif classes by performing abstract text-mining. Using machine learning models trained on the curated ELM resource dataset, each article is classified (i) to group novel articles with previously curated motif classes, (ii) to make suggestions that will simplify the manual classification/curation of these articles and (iii) to flag potential errors in the manual classifications.

In summary, the articles.ELM resource is a repository of SLiM literature that simplifies access to relevant SLiM information by shifting the burden of article discovery onto a computational annotation framework.

## Materials and Methods

### ELM training dataset

The classifiers were trained using the gold standard set of motif article:motif class pairs in the ELM database dataset. Each article in the ELM dataset describes functional modules that have been curated to one or more of the ELM classes. An ELM class describes a group of motifs that are functionally related, usually through binding the same pocket in a given protein or in a set of proteins with the same specificity determinants. We refer to this set of classified articles as the ‘ELM training dataset’. It comprises 2270 publications and 289 assigned ELM classes, linked as publication:ELM class pairs. The ‘ELM training dataset’ was retrieved from the ELM database on 1 March 2020 and will be updated to coincide with new releases of the ELM database.

### Manually curated test datasets

Several additional test datasets were used to analyse the ability of the classifier to correctly define a motif article from the article title and abstract. These datasets comprise articles about the following motifs (in parenthesis, the ELM classes mapped to the motif): the WW domain-binding PPxY motif (LIG_WW_1, LIG_WW_2, LIG_WW_3), the LC8-binding TQT motif (LIG_Dynein_DLC8_1), NLS motifs (TRG_NLS_Bipartite_1, TRG_NLS_MonoCore_2, TRG_NLS_MonoExtC_3, TRG_NLS_MonoExtN_4), Nuclear Export Signal (NES) motifs (TRG_NES_CRM1_1), the KEN box APC/C degron (DEG_APCC_KENBOX_2), the D box APC/C degron (DEG_APCC_DBOX_1), the PP1 phosphatase RVxF docking motif (DOC_PP1_RVXF_1), the Calcineurin phosphatase LxVP docking motif (DOC_PP2B_LxvP_1) and the Calcineurin phosphatase PxIxIT docking motif (DOC_PP2B_PxIxI_1). The datasets were manually curated over several years independently of the articles.ELM classifier project. The articles that were present in ELM and used to train the classifier were removed from the test datasets.

### HIPPIE interaction dataset

A dataset of protein interactions was retrieved from the HIPPIE database v2.2 on 14 February 2019 (1).

### Article annotation

The articles.ELM tool automatically annotates articles with a range of relevant biological data to allow in-depth search functionality. Article data including title, abstract, authors and publication details are retrieved from the PubMed REST services. The tool screens several protein resources for cross-references with PubMed to link protein data to the article. Articles are annotated with protein metadata based on PubMed cross-references with the following databases: (i) UniProt—manually curated articles linked to a UniProt entry (13); (ii) SciLite—text-mined UniProt linked biomolecule text mapping from the article (18); (iii) HIPPIE—protein–protein interaction data associated with an article (1); (iv) PDB—protein structural data associated with an article (19); and (v) ELM resource—manually curated short linear motif data (7). These resources annotate papers with protein-centric information and therefore we can link annotated papers from these resources that are also present in the ‘articles.ELM literature dataset’ to manually curated protein lists. All protein data in these resources are mapped to UniProt protein accessions (13). Consequently, the outputs of the cross-referencing pipeline for each protein-centric resource are PubMed identifiers mapped to UniProt protein accessions. The PubMed article-centric data for an article (e.g. title, abstract, authors and publication details) are then being augmented with protein metadata from UniProt (e.g. protein identifiers, protein names, gene names and their synonyms). These terms can then be used to search for relevant articles in the Search and Protein sections of the articles.ELM resource. Articles are also annotated with manually annotated terms describing the article content obtained from MeSH (12).

### Classification

The articles.ELM resource allows a user to take a set of labelled (classified) publications and use them to predict the class of an unlabelled (unclassified) publication. By applying supervised learning algorithms the classifier can learn from curated pairs of papers and assigned classes, and in return, provide a way to map an input (the title and abstract of an unknown publication) to an output (a motif class). The output of this procedure is the classification of the paper as referring (or not) to a certain ELM class. At release, the classifier is trained on the ‘ELM training dataset’.

The articles.ELM classifier methodology has five major parts.

#### Data preparation

The tool gathers the data required to train the machine learning classifier and classify articles from the PubMed REST service and stores the data locally in an XML format. The default information used as training data for the article classifiers and for classification by these classifiers are the article title and abstract.

#### TF-IDF document scoring

Relevant features are extracted from the retrieved articles by tokenizing the titles and abstracts into individual words, without punctuation and non-descriptive characters, collecting terms that serve as a descriptive summary of a publication’s content. Using the Natural Language Toolkit python library (2), the training data obtained from each publication are converted to a matrix of term-frequency times inverse-document-frequency (TF-IDF) word occurrences. The TF-IDF matrix encodes the enrichment of a term in a given document relative to the whole set of documents. For example, TF-IDF scores are high if a term occurs frequently in a document but not in the collection of documents. After TF-IDF transformation, common words are filtered and unigrams (terms made up of only one word) extracted for further consideration.

#### Classifier model construction

The TF-IDF vectors of each article are used to create a classifier based on the publication:classification pairs in the input training set. A linear SVM supervised model with SGD optimization is trained and tested using the scikit-learn Python library (8). The classifier allows the class of a novel document to be distinguished by calculating a metric analogous to a similarity score. The article similarity is quantified by a decision function ‘distance’ metric defined as the raw distance to the separating hyperplane for the classified article classes. This distance is therefore related to the similarity of the input article to the set of articles of a given class. The articles.ELM resource has an inbuilt option to benchmark the performance of the classifier. Benchmarking is run each time the datasets are updated and the results are made available automatically on the website at http://slim.icr.ac.uk/articles/benchmarking/.

#### Classifier significance distribution construction

A probability metric based on the distance score distribution is calculated for each class in the classifier. The articles describing protein–protein interactions from the HIPPIE database are used to calculate the background distance score distributions for each class. A ‘randomized article set’ is created by tokenizing the titles and abstracts of the HIPPIE articles (excluding articles used to train the classifier) and creating 1 million randomized titles and abstracts. The number of words in each randomized title and abstracts is set as the average number of words in a HIPPIE article title and abstracts. The ‘distance’ scores distribution is obtained by calculating the ‘distance’ scores of the articles in the ‘randomized article set’ against each class in the classifier. The distance scores can then be converted to a probability that represents the likelihood of seeing a given ‘distance’ score at random for each class. The article decision function distance distribution is converted to a cumulative probability and applied to each article during classification to provide an intuitive probabilistic classification metric to complement the stricter decision function distances.

#### Classification using the model

**Figure 1:**
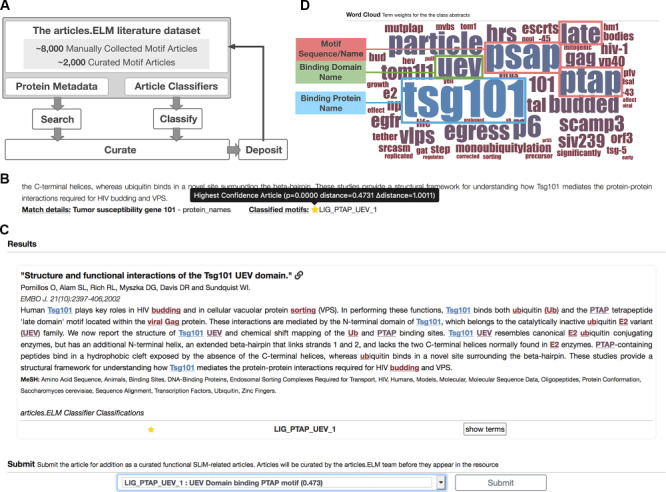
**Layout of the articles.ELM resource.** (**A**) Scheme of the articles.ELM framework. (**B**) Segment of the articles.ELM search page for the search term ‘Tumor susceptibility gene 101’ showing the source of the match as the protein names in the article metadata rather than the article title or abstract. The article also indicates motif classes classified for the article and, if available, any motifs curated in ELM or classified in articles.ELM for the article. (**C**) Segment of the articles.ELM classify page from the classification of the article titled ‘Structure and functional interactions of the Tsg101 UEV domain’ (11). The output shows the LIG_PTAP_UEV_1 motif class assigned to the article with high confidence (as marked by the full yellow star) and the article abstract is annotated with the key terms for the LIG_PTAP_UEV_1 classification. The terms are coloured by their weight and correspond to the colouring of the logo in the classified page (see panel D). The bottom half of the classify page allows the article to be manually annotated by the user using a list of motif classes and motif groups. (**D**) Segment of the articles.ELM classified page showing the word cloud representation of the classifier built on the ELM UEV domain-binding PTAP motif (ELM: LIG_PTAP_UEV_1) related articles from the ELM resource. The binding protein (TSG101), binding domain (the UEV domain) and motif sequence/name (PTAP, PSAP or Late domain motif) are highlighted here to demonstrate the relevance of the key terms.

The goal of the classifier is to identify the correct class of an unseen article. The articles.ELM classifier accepts an article for classification as a PubMed identifier. The article information is retrieved and processed as described for the construction of the classifier. The distance and probability measures of each article are returned. All classes with a distance probability score of less than 0.05, defined as significant classifications, are returned and the closest class is defined as the most likely classification for the article. In addition to the other measures, a ‘delta distance’ score is also calculated. The ‘delta distance’ is the raw distance minus the distance for a ‘probability’ cut-off of 0.0001 and is a useful metric for understanding the significance of articles:class pairs with a probability of 0 due to the sampled nature of the probability score calculation. The classifier also returns a list of high-weight keywords in the article that are strong discriminatory words for the classification (Figure 1).

## Results

### The articles.ELM resource

The articles.ELM resource is a community repository of protein motif literature (Figure 1A). The core of the articles.ELM resource is the ‘articles.ELM literature dataset’ and the ‘articles.ELM literature classifier’. The ‘articles.ELM literature dataset’ is a set of articles manually collected over the past decade for future curation in the ELM resource (7). At the time of submission, the ‘articles.ELM literature dataset’ contains 8811 manually collected articles, including 2270 publications from the ELM resource and 6541 motif articles awaiting curation in the ELM resource. The ELM resource groups motifs, and therefore articles describing these motifs, into classes dependent on shared function, specificity determinants and binding partners. The articles.ELM classifier is a text-mining tool to classify putative motif articles based on their similarity to these manually classified groups of articles in the ELM resource (7). Consequently, each class in the ELM resource has a corresponding class in the articles.ELM classifier. The articles.ELM text-mining tool uses a support vector machine (SVM) to classify articles based on the similarity of their titles and abstracts to those of the classified motif literature in the ELM resource (Figure 1B). The tool is used to annotate each article in the ‘articles.ELM literature dataset’ with predicted ELM classes.

### The articles.ELM resource website

The articles.ELM resource website provides an interface to explore the available motif literature in the articles.ELM literature dataset. Articles can be browsed based on manual and automated classification to motif classes or by filtering articles using search terms against the article abstract, title, authors and automatically annotated protein metadata. The website also allows members of the protein motif community to deposit motif related articles and submit manual classifications of articles. The resource is laid out in several sections to allow users to intuitively navigate the site. These sections are as follows.

#### Classes

The articles.ELM resource ‘classes’ page provides a list of the class names and a short one line description for each class within the articles.ELM resource. The ‘classes’ page acts as an index for the resource and gives an overview of data stored in the resource.

#### Search

The articles.ELM resource allows all articles in the ‘articles.ELM literature dataset’ to be searched using their annotated data. Basic searching includes the article abstract, title and authors. Each article is augmented with searchable protein metadata from selected protein databases using their PubMed cross-references to improve the article findability (see Materials and Methods). Searchable protein metadata annotation includes protein names, gene names or Universal Protein Resource (UniProt) accessions related to the articles. For example, a search for ‘Tumor susceptibility gene 101’ can return articles that do not have that term in the article title or abstract if at least one of the metadata sources has protein data related to the TSG101 protein for the article (Figure 1B). These protein search terms are expanded using UniProt synonyms. Complex searches can be built using the optional search options (AND, OR, Exact searches). The results of the search include the identity of the data attribute that matches the search term. This can include title, abstract, author or protein metadata. When the match is against the protein metadata, the source of protein metadata is given.

#### Browse

The articles.ELM resource allows all articles in the ‘articles.ELM literature dataset’ to be browsed by class through the ‘browse’ page. Each article in the ‘browse’ page is annotated with the source of the classification as follows: ELM, an article annotated to the class by the ELM consortium; Candidate, an article annotated as a candidate article for the class by the articles.ELM team or contributors; and Classified, an article annotated as an article for the class by the articles.ELM text-mining classification tool.

#### Proteins

The articles.ELM resource allows all protein metadata cross-referenced to the articles.ELM literature dataset (see Materials and Methods) to be directly searched or browsed through the proteins page. Protein metadata for each article is retrieved by cross-referencing with manually curated data linking a protein to an article from PubMed, UniProt, ELM, Protein Data Bank (PDB) and interaction datasets. Searches can find exact matches to UniProt accessions (e.g. ‘P06400’), protein or gene names (‘Retinoblastoma’ or ‘RB1’) and any common synonyms (‘pRB’, ‘pp110’). A link is provided to view all articles for the selected protein, displayed similarly to the search results page.

#### Candidates

The ‘candidates’ page lists all manual classifications submitted to the resource by the articles.ELM team or community members through the ‘submit’ page.

#### Classified

The ‘classified’ page of the resource provides access to the automatically classified articles of the ‘articles.ELM literature dataset’ grouped using the ELM class classifications. The classified articles set contains the significant articles (*P* < 0.05) returned by the articles.ELM text-mining tool for each ELM class. The top of the page consists of a word cloud displaying the relative weights of the terms for the article classifier of the class (Figure 1D). The remainder of the page contains significant article matches and their title, article details and abstract. Articles are ranked by their classifier distance score, a measure of similarity to the curated ELM class. The abstract of each article is colour-coded by the classifier word weighting using the same colour palette as the word cloud. Articles curated in the ELM database and therefore used in the classifier training can be hidden to show only novel uncurated motif articles.

#### Submit

Novel motif articles are deposited through the ‘submit’ page. The ‘submit’ page includes a classification dropdown menu that allows a user to manually assign an existing class to an article and submit that annotation to the articles.ELM resource. Candidates articles can also be tagged as belonging to a ‘Novel Class’ of motif if the article cannot be classified as an ELM motif class. Novel motif-related articles can be added to the resource by any user. Curation groups and papers groups are also available to annotate papers to larger less specific motif categories. Several curation drives options are also available for ongoing curation projects (e.g. ‘Viral Motifs’). The user-classified articles will be periodically reviewed by the articles.ELM team to improve the quality of the articles.ELM classifiers.

#### Benchmarks

The articles.ELM resource benchmarks the performance of the classifier and annotation tools each time the datasets or classifiers are updated. Updated benchmarking results are made available automatically on the ‘benchmarks’ page.

#### Downloads

Public releases of the database can be downloaded in JSON and tab-delimited text formats from the ‘downloads’ page allowing easy computational parsing and compatibility with commonly used spreadsheet applications. Article information can also be downloaded in JSON and tab-delimited text formats from the ‘browse’, ‘search’, ‘classified’ and ‘candidates’ pages to allow access to specific subsets of the data stored by the articles.ELM resource.

#### Classification and curation infographics

The articles.ELM resource uses a consistent scheme throughout the website to denote the classification and curation status of an article.

#### Classifier scoring and infographics

The classification confidence of the match to a class is clearly indicated using a simple star-based infographic system that denotes the quality of the classifier match. The raw classifier scores for the article are displayed by hovering over each star. The classifier scores each article with three metrics for each class: ‘distance’, ‘probability’ and ‘delta distance’ (see Materials and Methods). As a rule of thumb, a positive ‘distance’ score for a class denotes a ‘classified’ abstract and would represent articles that are highly similar to a particular class. Conversely, a negative ‘distance score’ for a class represents an article that has not been ‘classified’ to a given class. Empirically, the closer a negative ‘distance score’ is to zero the better the match to the motif class. As the articles.ELM resource is exploratory in nature, the resource does not have a strict requirement for a positive ‘distance’ score for the returned predicted classes. Instead, the resource annotates each article with a list of the classes with a ‘probability’ score above 0.05. This ‘probability’ score defines the likelihood of seeing an abstract with an observed level of similarity to a given class by chance (see Materials and Methods).

#### Curation source and infographics

Articles that have been curated or classified are marked with tags and the colouring of the tag relates to the source of curation or classification: grey ‘ELM article’ tags denote articles curated in the ELM resource; blue ‘Candidate article’ tags are given to articles classified in articles.ELM; on the class-centric pages, a teal ‘Candidate article’ tags denotes articles classified in articles.ELM but to a different class; and green ‘Submit’ tags mark articles not currently curated or classified. Clicking on a green tag links to the ‘submit’ page allowing the user to manually classify the article.

### Benchmarking

#### ELM dataset reannotation

Protein/gene name-centric searches are common use cases for the articles.ELM resource. Consequently, the articles in ‘articles.ELM literature dataset’ are supplemented with protein metadata (see Materials and Methods) to improve article searchability for protein/gene name-centric searches. The ELM dataset reannotation analysis is presented to show that the addition of protein metadata for an article from external resources can correctly annotate UniProt accessions of the motif-containing or binding proteins in manually curated motif articles from the ELM resource. This suggests that the addition of protein metadata will improve the quality of text searches using protein or gene names. Importantly, the addition of protein metadata will improve results even when the protein name is not present in the title or abstract. Furthermore, the addition of synonyms of these protein or gene names as protein metadata for an article will improve search results even when a non-canonical name is used.

**Figure 2:**
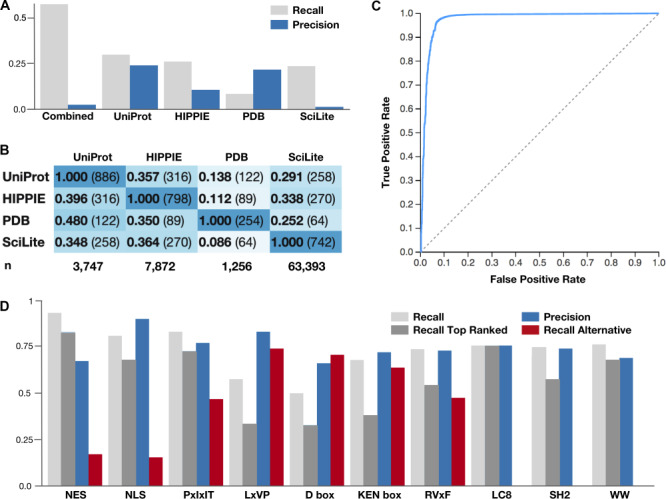
**Benchmarking results for the sources of article protein annotation and classification.** (**A**) The ability of each source of article protein annotation to correctly reannotate the proteins manually curated for motif articles by the ELM resource. Recall is the fraction of UniProt accessions annotated for the articles in the ELM resource that were returned. Precision is the fraction of UniProt accessions returned for the ELM resource by each source of article protein annotation that are correct. (**B**) The overlap of the correctly identified UniProt accessions between each article annotation resource. The denominator of the proportion relates to the row. (**C**) ROC curve for the 5-fold cross-validation of the ELM class annotation of the ELM training set. Scores are calculated for a single fold scoring set against classifiers trained with the four remaining folds as a training set. Data describe a binary classification pooling the negative classes for each class in the classifier. (**D**) The ability of the article.ELM classifier to identify the correct ELM class in 10 manually curated, real-world motif article datasets. Recall and precision are shown, along with the ability of the classifier to recognize the curated motif class as the top-scoring class (Recall Top Ranked) and its ability to recognize a related, alternative motif (Recall Alternative). A detailed and up-to-date version of the data shown here is available at http://slim.icr.ac.uk/articles/benchmarking/.

Articles from the ELM resource were annotated with UniProt accessions using PubMed cross-referenced protein metadata from UniProt, SciLite, Human Integrated Protein–Protein Interaction rEference (HIPPIE) and PDB (see Materials and Methods). The proportion of proteins manually curated to contain a motif that could be reannotated by external cross-referencing was calculated for the articles of the ELM resource. No single source of annotation data could comprehensively reannotate the ELM resource article dataset (Figure 2A). Protein annotation through PubMed cross-referencing from UniProt curated data returned the correct protein for 27.9% of the articles and HIPPIE interaction data and SciLite returned the correct protein for ~25% of the articles. Mapping through PDB was limited in terms of coverage (low recall of 8.0%); however, when structure-linked protein data were available it often permitted annotation of the correct protein (20.2% precision comparable with the best data source UniProt at 23.6%).

Overall, the precision was low and many of the proteins annotated for an article were not present in the ELM resource. However, the disappointing performance was not surprising as the test was only performed for the motif-containing protein and not the motif-binding partner as these data are only partially curated in the ELM resource. One outlier in terms of precision is SciLite that returned a large number of alternative annotations that are not related to the annotated ELM protein. SciLite has a precision of 1.2% compared with 23.6% for UniProt, 20.2% for PDB and 10.1% for HIPPIE. Upon closer inspection, many of these SciLite annotations were mapped to the incorrect species. This suggests that when the species is known, annotation transfer by homology in the SciLite annotation framework could improve the accuracy of these annotations. Furthermore, SciLite mapped significantly more proteins for each article. For the ELM articles, SciLite mapped 63 393 proteins compared with 3747 for UniProt, 7872 for HIPPIE and 1256 for PDB.

Interestingly, there was limited overlap between the correctly identified UniProt accessions from each source of article annotation data (Figure 2B). Consequently, by combining the sources of data, the resource can correctly annotate the proteins at a much higher rate than any single data source alone with a recall of 55.6% for the four sources of metadata combined compared with 27.9% for UniProt, the best single source (Figure 2A). Consequently, the data suggest that using combined protein metadata in article searches improves access to the motif literature over any one data source alone. Therefore, the article.ELM resource annotates the ‘articles.ELM literature dataset’ with protein metadata from UniProt, SciLite, HIPPIE and PDB to improve the search results for protein/gene name-centric searches.

#### 5-fold cross-validation of the ELM class annotation

The ability of the articles.ELM classifier to correctly classify motif articles of the ELM dataset was tested using a 5-fold cross-validation benchmark protocol. The classifier used in articles. ELM is a linear SVM supervised model with stochastic gradient descent (SGD) optimization. The model was trained and tested using the article titles and abstracts. In total, there were sufficient data to perform the 5-fold cross-validation benchmark on 1483 articles. The analysis tested 339 240 article:class pairs of which 2280 are significant at a *P*-value cut-off of 0.05. Of the 2280 significant classifications, 1113 were classified to the correct motif class. The remaining 1167 classifications were incorrect; however, a considerable subset were assigned to classes from the same family of motif as the correct classification. The classifier performed with a precision of 0.488, a true positive rate (recall) of 0.751 and a false positive rate (fallout) of 0.003. The method achieved an AUC of 0.978 (Figure 2C). The average distance score ‘probability’ of true article:class pairs was 0.079 compared with 0.652 for the background article:class pairs.

#### ELM class annotation of a manually curated dataset

Ten sets of manually curated motif articles were created to benchmark the classifier (see ‘Manually curated test datasets’ in Materials and Methods). The articles in these sets described an instance of a motif class present in the ELM resource where the article itself was not annotated in the ELM resource. The datasets were created across several projects independently of the classification tool of the articles.ELM resource using simple literature searching and manual classification. The articles.ELM classifier was applied to the articles and the classifications were quantified to determine the ability of the classifier to identify the correct ELM class of large real-world curated datasets (Figure 2D).

In total, 822 article:class pairs were curated for the benchmark set. Of the 1466 significant predictions produced by the classifier, 1104 were for the correct curated motif class, with 545 as the top-scoring class. These numbers are larger than the number of articles as a given class can have more than one ELM classification; for example, the NLS dataset maps to ELM motif classes TRG_NLS_Bipartite_1, TRG_NLS_MonoCore_2, TRG_NLS_MonoExtC_3 and TRG_NLS_MonoExtN_4. The benchmarking set had a mean recall of 0.75 and a mean precision of 0.76. The performance was generally good with the exception of the LxVP, D box and KEN box datasets in which <50% of the articles were correctly identified as significant and the correct class was recognized as the top-scoring classification. In these cases, the motif classes generally co-occur in the same protein, or bind to different pockets on the same motif-binding protein, as an alternative distinct motif class. For example, the D box and KEN box motif classes both bind to the same subunit of the anaphase-promoting complex or cyclosome (APC/C) (4). For the seven test sets with related alternative motif classifications, representing a grey area in terms of false positives, 202 of the significant classifications were for the alternative motif.

It should be noted that for benchmarks such as the SH2 dataset many of the abstracts will contain a specific term (e.g. ‘SH2’) and these articles could easily be classified by a simple rule-based classifier. In reality, such cases are artificially easy problems. However, the advantage of the articles.ELM classifier is that it does not require a single manually curated term or a set of search terms. Instead, the classifier relies on all the terms in the title and abstract of a set of previously characterized articles and the relative occurrence of these terms in the articles of the motif class against the rest of the articles in the dataset. Consequently, other test cases, such as the PP1 phosphatase and APC/C E3 ligase degron motifs datasets, can be correctly classified with more complex combinations of terms. Furthermore, it should be noted that even for the SH2 dataset, it is not always the case that SH2 is in the title or abstract.

#### Highly weighted terms in ELM class classifier

For a classifier that is correctly describing a set of articles from a given class, the heavily weighted terms should be related to the biology of the class. The motif classifier term weightings (see Figure 1D) for each class were investigated to understand their relationship to the motif class they describe. For each motif class, the top 5 terms were manually curated to define terms that relate to the correct binding partner (gene or protein name) for the motif, the correct common name or consensus of the motif, the localization for targeting motifs and the modification for modification motifs. Upon analysing the top 5 weighted terms for each class of the 289 classes using these curated terms, the correct binding partner was in the top 5 weighted terms for 165 classes, the correct common name or consensus of the motif for 60 classes, the correct binding domain for 33 classes, the correct modification for 15 classes and localization for 12 classes. Only 79 classifiers did not have a classifier term related to the motif in the top 5 terms. Of the 1350 top five weighted terms, 429 (29.6%) are clearly related to the motif, motif binding partner or motif function. The remainder is largely related to the motif-containing proteins. A full list of the top 5 weighted terms for each motif class classifier is available at http://slim.icr.ac.uk/articles/benchmarking/?benchmark_type=classifier_benchmarking_keywords.

## Discussion

The rate of production of protein motif data far outpaces the rate of curation and annotation. It is estimated that only ~5% of human SLiMs have been characterized, and interest in the field is increasing year on year (14). Consequently, the curation gap between the articles that are available and the articles that have been curated will only increase over the coming years unless adequate funding is made available.

We have developed a computational framework to deposit, search, classify and annotate literature related to SLiMs. This automated classification and annotation framework will promote the exchange and use of SLiM literature by indexing the data in the accessible and searchable articles.ELM resource. Such tools are key to unlocking the potential of the currently available scientific literature to classify existing motifs, discover new motif instances and direct future research. Furthermore, articles.ELM provides a blueprint for similar efforts in other biological fields with similar curation bottleneck issues.

The classifications can also help to accelerate the curation of the ELM database by providing pre-classified papers to keep motif annotations up to date. The articles.ELM resource is already cross-linked with the ELM resource allowing ELM users to access this extended collection of motif literature. The repository of article data from the resource not only is updated and classified by the articles.ELM team but also allows the community to provide novel articles and classifications. We hope that the motif community will contribute to the resource. We are particularly interested in direct submission of the research output of experimental motif researchers. We anticipate that the articles.ELM resource will be widely used and integrated into the data resource infrastructure of the protein motif community.
